# Transient swallowing‐induced atrial tachycardia in a patient with genotyped hypertrophic cardiomyopathy

**DOI:** 10.1002/ccr3.3015

**Published:** 2020-06-08

**Authors:** Noboru Fujino, Kenshi Hayashi, Kenji Sakata, Hayato Tada, Chiaki Nakanishi, Toyonobu Tsuda, Akihiro Nomura, Shohei Yoshida, Yuichiro Sakamoto, Takeshi Kato, Hiroshi Furusho, Masa‐aki Kawashiri, Masayuki Takamura

**Affiliations:** ^1^ Department of Cardiovascular Medicine Kanazawa University Graduate School of Medical Sciences Kanazawa Japan; ^2^ Division of Cardiology Toyohashi Heart Center Toyohashi Japan

**Keywords:** cardiac beta‐myosin heavy chain gene, hypertrophic cardiomyopathy, mutation, swallowing‐induced atrial tachycardia

## Abstract

Most cases of swallowing‐induced atrial tachycardia require radiofrequency catheter ablation for a permanent cure; however, the arrhythmia subsided after temporary prescription of verapamil in a patient with genotyped hypertrophic cardiomyopathy.

## Graphical abstract

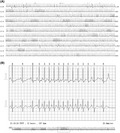

## 1 INTRODUCTION

Swallowing‐induced atrial tachycardia is an uncommon condition defined as supraventricular tachycardia occurring during a swallow. Most cases of this arrhythmia require radiofrequency catheter ablation for a permanent cure. In the present case, however, swallowing‐induced atrial tachycardia subsided after temporary prescription of verapamil in a 42‐year‐old woman with genotyped hypertrophic cardiomyopathy.

Swallowing‐induced atrial tachycardia (SIAT) is a rare phenomenon.^1^ SIAT was first reported in 1926^2^ and is defined as tachyarrhythmia that occurs reproducibly and consistently during a swallow.^1^, ^2^, ^3^, ^4^, ^5^, ^6^, ^7^, ^8^ Tachyarrhythmias induced by a swallow include premature atrial contraction, atrial tachycardia, and atrial fibrillation. Although patients with SIAT develop palpitations triggered by deglutition, most cases are not associated with structural heart disease, and successful treatment of SIAT with radiofrequency catheter ablation (RFCA) or pharmacological drugs has been described.^1^, ^2^, ^3^, ^4^, ^5^, ^6^, ^7^ A recent report described an adolescent patient with hypertrophic cardiomyopathy (HCM) who developed SIAT and was treated by RFCA.^8^ We herein report a case of SIAT in a patient with HCM caused by p.Phe244Leu in the cardiac beta‐myosin heavy chain gene (*MYH7*). The patient had recurrent palpitation attacks provoked by deglutition and was diagnosed with SIAT. We prescribed oral verapamil, and her symptoms gradually became less frequent during the next 3 months before subsiding completely. After discontinuing verapamil, 24‐hours Holter electrocardiogram (ECG) monitoring showed no SIAT. The patient has remained free from SIAT for >10 years without the requirement for medication.

## 2 CASE PRESENTATION

**FIGURE 1:**
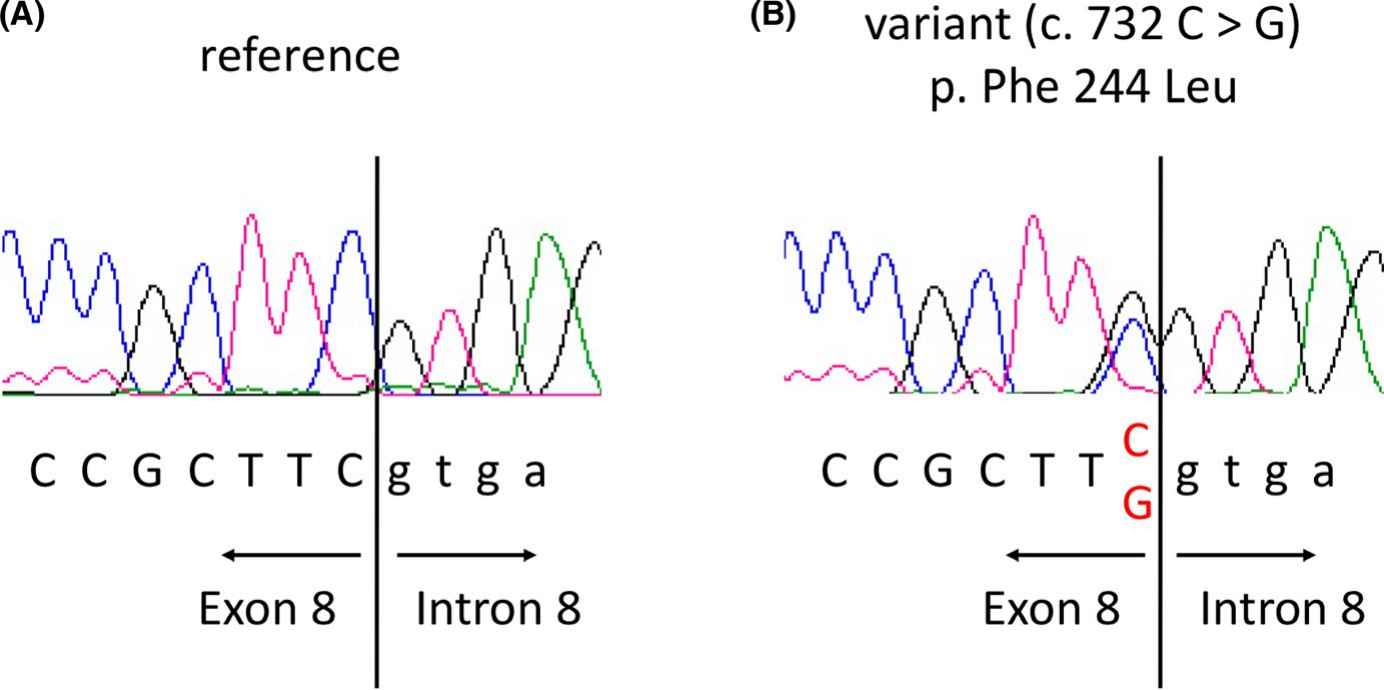
Cardiac beta‐myosin heavy chain gene (*MYH7*) variant identified in a patient with swallowing‐induced atrial tachycardia and hypertrophic cardiomyopathy. A, Sequence trace of the reference. B, Sequence trace of the *MYH7* variant p.Phe244Leu caused by c.732 C > G (hetero) in the patient

We herein describe a 42‐year‐old woman with HCM caused by p.Phe244Leu in *MYH7* who presented with an approximately 1‐week history of recurrent palpitations while eating. At 40 years of age, she had visited our hospital for a consultation regarding her condition because her mother had developed a cerebral infarction caused by atrial fibrillation and HCM. The family history showed that her uncle (the brother of her mother) had severe heart disease (details unknown) and died suddenly in his early 40s. An ECG at that time showed sinus rhythm with ST‐T changes suggesting left ventricular hypertrophy. Echocardiography revealed localized mild hypertrophy of the interventricular septum with no findings of left ventricular outflow tract obstruction. The patient requested genetic analysis of HCM (approved by the Bioethical Committee on Medical Research, Kanazawa University, and with written informed consent), and we detected p.Phe244Leu in *MYH7* in both the patient and her mother (Figure 1).

**FIGURE 2:**
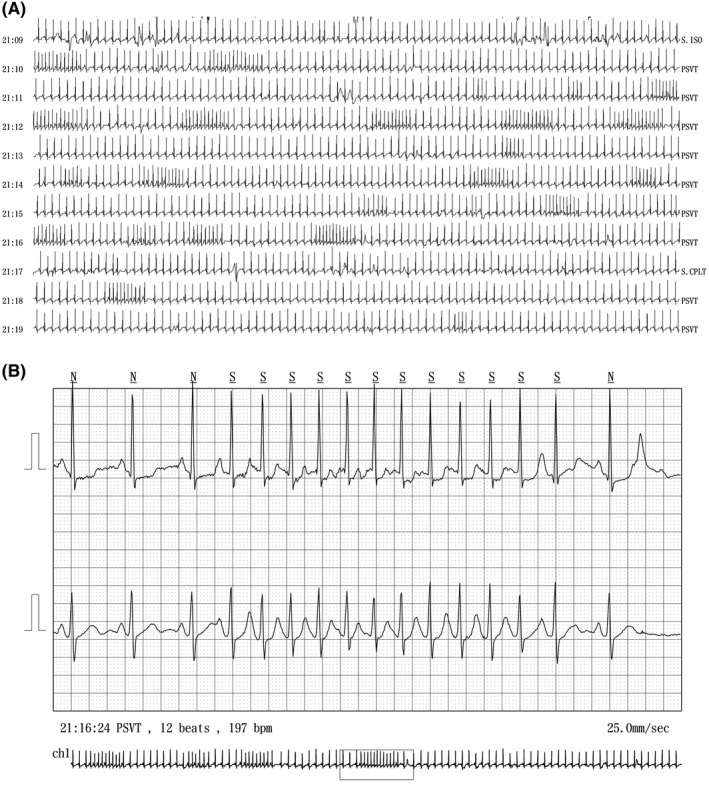
A 24‐h Holter electrocardiogram (ECG) readout of a recording obtained during dinner. A, Holter ECG recording from 21:09 to 21:19. B, Holter ECG recording at 21:16 (enlarged version of A)

**FIGURE 3:**
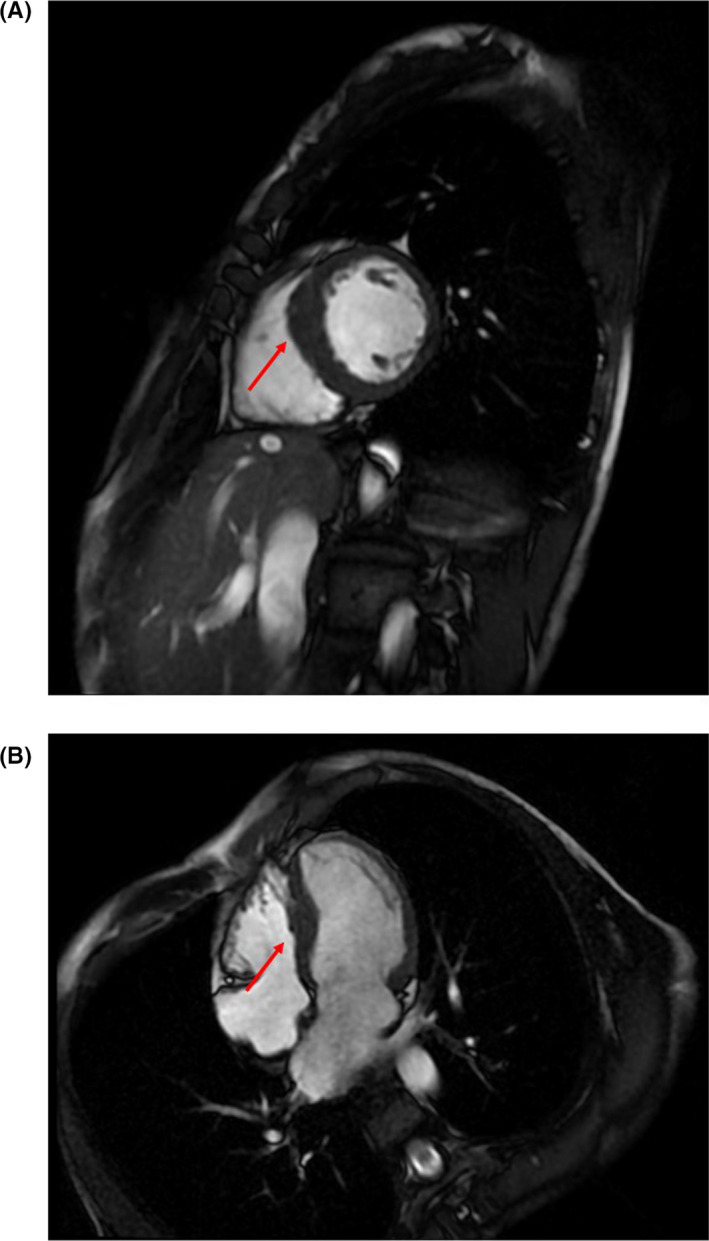
Cine magnetic resonance imaging of the patient with swallowing‐induced atrial tachycardia and hypertrophic cardiomyopathy. A, The short‐axis view shows localized mild hypertrophy of the interventricular septum (red arrow). B, The four‐chamber view also shows localized mild hypertrophy of the interventricular septum (red arrow)

We then examined the patient every 3 months, although she was asymptomatic and therefore required no medication. At 42 years of age, however, she reported episodic palpitations that occurred while eating. She also had a near‐syncopal feeling in the first few days of the onset of palpitations. A physical examination was unremarkable, and her baseline thyroid function was within normal limits. An ECG and echocardiographic examination revealed findings similar to those of her prior examinations, including her left atrial size. The interventricular septal wall dimension was 13 mm. A 24‐hours Holter ECG recording revealed brief runs of atrial tachycardia temporally associated with her symptoms during swallowing (Figure 2). The tachycardia lasted 5‐29 beats, while the ventricular rate varied from 150 to 200 beats per minute. The tachycardia occurred with every episode of swallowing and then spontaneously reverted to sinus rhythm. Upper gastrointestinal endoscopy findings were normal. Cardiac magnetic resonance imaging of the chest revealed localized mild hypertrophy of the interventricular septum, but no tumors or other abnormalities, especially near the heart (Figure 3). There were no findings of late gadolinium enhancement. Thus, we diagnosed the arrhythmia as SIAT.

We followed her tachycardia with no drug treatment for 2 weeks, and her symptoms did not improve. Thus, we prescribed verapamil (40 mg orally, three times per day) because of its safety profile. Her symptoms did not change for 2 months, but they gradually became less frequent during the next month before subsiding completely. We discontinued the verapamil and performed 24‐hours Holter ECG monitoring, which revealed no SIAT. Furthermore, no SIAT was observed on Holter ECG monitoring performed at 1, 2, 3, 6, and 7 years after the first SIAT event. At the 10‐year follow‐up, the patient was still free from any SIAT without medication.

## 3 DISCUSSION

Swallowing‐induced atrial tachycardia is a rare disorder that was first described by Sakai and Mori^2^ in 1926 as “Schlucktachycardie”.^1^, ^2^ The arrhythmia is predominantly detected in adults without structural heart disease. Although it is rare, numerous cases have been reported worldwide.^1^, ^2^, ^3^, ^4^, ^5^, ^6^, ^7^, ^8^ The events that provoke this transient supraventricular tachyarrhythmia include swallowing, coughing, and mechanical stimulation of the esophagus.^1^, ^2^, ^3^, ^4^, ^5^, ^6^, ^7^, ^8^ In most cases, the tachycardia is self‐limiting with spontaneous termination within a few seconds of onset. One study showed that the prevalence of SIAT was 0.6%.^1^ In that study, the arrhythmia was observed in three men among 544 patients who underwent an electrophysiologic study and RFCA for symptomatic premature atrial contractions, paroxysmal atrial tachycardia, and/or paroxysmal atrial fibrillation. Of the 40 reviewed cases, the mean age at symptom onset was 49 years and male predominance was shown.^1^

Swallowing‐induced atrial tachycardia is not usually associated with structural heart disease; in the present case, however, it occurred in a 42‐year‐old woman with HCM caused by p.Phe244Leu in *MYH7*. A case of SIAT in a 17‐year‐old man with HCM treated by RFCA was also recently reported.^8^ These are the only reports of SIAT detected in patients with HCM, and our report is the first to describe SIAT in a patient with genotyped HCM. A comprehensive study showed that only three patients had structural heart disease (2 with old myocardial infarctions and 1 with mitral valve prolapse) among 40 patients with SIAT.^1^ This suggests the rarity of SIAT with structural heart disease, and our manuscript is a valuable addition to the literature. HCM is an autosomal dominant inherited myocardial disorder with various clinical characteristics and has a prevalence of up to 1 in 500 individuals.^9^ The *MYH7* and myosin‐binding protein C genes, which encode sarcomere proteins, are considered the main causal genes of HCM. The mutation is located in the nucleotide‐binding pocket of the globular head of myosin.^10^ Because HCM is a progressive disorder that is sometimes complicated by left ventricular systolic dysfunction or lethal arrhythmia,^9^ we repeated the echocardiography examination after the onset of SIAT in our patient. However, the findings, including those of the left atrium, were very similar to those 2 years prior. Cardiac magnetic resonance imaging showed only mild hypertrophy of the interventricular septum, and no other abnormalities were detected around the heart. Because the etiology of SIAT remained unclear, we followed the patient for 2 weeks without drug treatment. However, her symptoms did not improve.

Several reports have described successful control of arrhythmia following treatment with drugs such as class Ia antiarrhythmics and verapamil.^1^, ^3^, ^5^, ^7^ For example, two patients reportedly showed no recurrence of SIAT after 5‐9 months of treatment with verapamil + quinidine sulfate and verapamil + disopyramide, respectively, representing very rare courses of this disease.^3^, ^7^ In the first study, a 64‐year‐old man with SIAT was treated with a combination of verapamil (320 mg/d) and quinidine sulfate (1200 mg/d) over 5 months with a marked improvement in palpitations. He thereafter discontinued these medications and noted resolution of his symptoms. In the second study, a 38‐year‐old woman with deglutition‐induced atrial fibrillation was treated with a sustained‐release formulation of disopyramide at 100 mg twice a day, which was subsequently increased to 200 mg twice a day. These treatments resulted in partial resolution of the arrhythmia, and the addition of 180 mg of verapamil each day led to a complete resolution of symptoms. Over a 9‐month period, the patient was weaned from both medications. She was still symptom‐free 5 years after those episodes. In those studies, the authors speculated that the medications induced spontaneous remission of SIAT.^3^, ^7^ We also prescribed verapamil for 3 months in the present case. We did not try to prescribe other medications such as beta‐blockers, class Ia antiarrhythmics, angiotensin‐converting enzyme inhibitors, or angiotensin receptor blockers. Our patient's symptoms did not change for first 2 months; however, they gradually became less frequent during the next month before subsiding completely. Because verapamil is a rapidly acting drug, it is difficult to conclude that verapamil directly alleviated the SIAT; rather, spontaneous remission of SIAT might have occurred during the 3‐month treatment with verapamil.

There are two potential mechanisms of SIAT. One involves mechanical stimulation of the left atrium as liquids and/or solids pass through the esophagus, while the other involves a neural reflex originating in the esophagus.^1^, ^2^, ^3^, ^4^, ^5^, ^6^, ^7^, ^8^ Kalloor et al^6^ reported that SIAT may be induced by mechanical stimulation of the esophagus. However, the origin of SIAT is not adjacent to the esophagus in all cases.^1^ Vagal stimulation is also implicated in the development of SIAT.^1^, ^3^, ^5^, ^7^ Furthermore, there is evidence that RFCA of the ganglionated plexus or a site close to this plexus is effective in patients with SIAT,^4^, ^8^ suggesting a relationship between SIAT and the vagal reflex. Given these findings, we speculate that the SIAT in our case subsided on its own along with improvement in the patient's vagal status. If our patient develops recurrence of SIAT in the future, we will consider an electrophysiological study and RFCA, as previously reported.^1^, ^4^, ^8^

In conclusion, SIAT is a rare disease, and the long‐term consequences after medication remain unclear. The unusual case of SIAT in a patient with genotyped HCM described in the present report provides new insights into its pathogenesis. Thus, medication or conservative therapy might be considered before performing RFCA in patients with SIAT.

## CONFLICT OF INTEREST

The authors have no conflicts of interest to disclose.

## AUTHORS’ CONTRIBUTIONS

NF, KH, and YS: treated the patient and were responsible for collection and interpretation of the data and drafting of the manuscript. KS, HT, CN, TT, AN, SY, TK, HF, MK, and MT: helped to interpret the data and revise the paper.
